# Muscle-Specific Mis-Splicing and Heart Disease Exemplified by RBM20

**DOI:** 10.3390/genes9010018

**Published:** 2018-01-05

**Authors:** Maimaiti Rexiati, Mingming Sun, Wei Guo

**Affiliations:** 1 Animal Science, University of Wyoming, Laramie, WY 82071, USA; rmaimait@uwyo.edu (M.R.); msun2@uwyo.edu (M.S.); 2 Center for Cardiovascular Research and integrative medicine, University of Wyoming, Laramie, WY 82071, USA

**Keywords:** alternative splicing, muscle-specific splicing factor, heart disease, RNA-binding motif 20, titin

## Abstract

Alternative splicing is an essential post-transcriptional process to generate multiple functional RNAs or proteins from a single transcript. Progress in RNA biology has led to a better understanding of muscle-specific RNA splicing in heart disease. The recent discovery of the muscle-specific splicing factor RNA-binding motif 20 (RBM20) not only provided great insights into the general alternative splicing mechanism but also demonstrated molecular mechanism of how this splicing factor is associated with dilated cardiomyopathy. Here, we review our current knowledge of muscle-specific splicing factors and heart disease, with an emphasis on RBM20 and its targets, RBM20-dependent alternative splicing mechanism, RBM20 disease origin in induced Pluripotent Stem Cells (iPSCs), and RBM20 mutations in dilated cardiomyopathy. In the end, we will discuss the multifunctional role of RBM20 and manipulation of RBM20 as a potential therapeutic target for heart disease.

## 1. Introduction

Alternative splicing is a molecular process by which introns are removed from pre-mRNA, while exons are linked together to encode for different protein products in various tissues [1]. It is a crucial mechanism for modulating expression of genes in multiple cellular processes and the major contributor to proteomic diversity [2]. In humans, nearly 94% of all genes undergo alternative splicing leading to approximately four times more proteins expressed than genes in human genome [3,4]. Splicing is conducted by the spliceosome, a large complex of ribonucleoprotein comprised of four small nuclear ribonucleoproteins (U1, U2, U4/U6 and U5 small nuclear ribonucleoproteins (snRNPs)) and other non snRNP splicing factors [5,6,7]. Whether an exon is removed or included from a pre-mRNA depends on splicing signals from RNA sequence and regulatory elements [7]. First, splicing needs proper recognition of exon-intron boundaries and exon-intron sequence elements by spliceosomes. The most common sequence elements at exon-intron junctions are 5’ and 3’ splice sites [7,8]. In addition, splicing is regulated by *cis*-regulatory elements, which include exonic splicing enhancers (ESEs), exonic splicing silencers (ESSs), intronic splicing enhancers (ISEs) and intronic splicing silencers (ISSs). Enhancer elements mainly participate in constitutive splicing, while silencers have a dominant role in alternative splicing. *Cis*-regulatory elements function as enhancers or silencers according to their position and context to recruit *trans*-regulatory factors, which consist of RNA binding proteins (RBPs) and other splicing factors [7,9].

It is now established that abnormal alternative splicing contributes to a large number of human diseases [10]. Mutations in *cis*-regulatory elements, *trans*-regulatory factors and other components of the spliceosomes cause several disorders in humans [11,12]. According to the Human Gene Mutation Database about 3700 mutations are implicated with splice sites, which affects pre-mRNA splicing [12,13]. The mutations cause gain or loss of splicing function and alternation in splicing factors which could trigger the onset of various diseases, for example, heart disease. In cardiomyopathy, abnormal splicing of sarcomeric and ion channel genes have been reported in several studies. These changes can ultimately alter the normal internal architecture and homeostasis of the heart [14,15]. For example, previous literature reported that upregulated expression of splicing factor splicing factor 3B subunit 1 (SF3B1) causes mis-splicing of ketohexokinase and triggers the onset of cardiac hypertrophy [16]. Also, an imbalance between splicing factors CUG-binding protein 1 (CUGBP1) and Musleblind-like protein 1 (MBNL1) causes myotonic dystrophy as a result of alternative splicing of troponin T in the heart [17]. Recently, it was reported that RNA-binding motif 20 (RBM20) regulates muscle-specific gene splicing such as titin splicing [18,19,20]. Deficiency of Rbm20 in animal models leads to the development of dilated cardiomyopathy (DCM) and mutations in RBM20 have been identified in human patients with DCM [18,21,22,23]. It is crucial to understand the molecular and cellular mechanisms of the splicing alternation during disease onset and development, which provides novel insight into the molecular therapy for heart disease. In this review, we will focus on the intersection between muscle-specific alternative splicing and heart disease, with a particular emphasis on a muscle-specific splicing factor RBM20 and its regulatory roles in heart disease.

## 2. Muscle-Specific Splicing Factors and Heart Disease

Striated muscles are one of the highly specialized tissues that undergo tissue-specific alternative splicing events [24,25]. A sarcomere, the most basic contractile component of striated muscle, consists of contractile, regulatory, cytoskeleton and other essential proteins [26,27,28]. Sarcomeric genes undergo dynamic alternative splicing events, which are mainly regulated by muscle-specific splicing factors [15,29]. Until now, well studied muscle-specific splicing factors include MBNL1, CUGBP1, RNA-binding motif protein 24 (RBM24), RNA-binding protein fox-1 homolog 1 (RBFOX1) and RBM20 (Table 1). These splicing factors are highly expressed in striated muscles and orchestrate muscle-specific alternative splicing events. Mutations and dysregulation of these splicing factors have been identified in various human heart disease, such as hypertrophic and dilated cardiomyopathy [14,29].

Myotonic dystrophy (DM) is the best-known splicing-associated neuromuscular disease which is characterized by muscle weakness, cardiac conduction abnormalities, and cardiomyopathy [35,36,37]. Cardiac abnormalities are common in more than 80% of the DM patients and related to 30% of the mortality in DM caused death [38,39]. Dysregulation of splicing factors MBNL1 and CUGBP1 are involved in the disease process. The muscle-specific splicing factor MBNL1, controls alternative splicing of exon 5 in cardiac troponin T, and it competes with splicing factor U2 snRNP auxiliary factor 65-kDa subunit (U2AF65) for the binding site at the 3′ of troponin T intron [40]. In the disease state, the extended CUG repeats in the RNA sequence sequester MBNL1 and impair its splicing activity in the heart and skeletal muscle. Both loss of function of MBNL1 and gain-of-function of CUGBP1 can trigger the development of the DM-like phenotype [30,31,32]. Mouse model overexpressing CUGBP1 is sufficient to induce the heart-specific DM phenotype [33,34]. Even though the underlying mechanism for the DM-dependent cardiac abnormalities is not well understood, it is possibly related to the dysregulation of splicing activity of MBNL1 and CUGBP1, which are responsible for splicing of Troponin T2 (TNNT2), and LIM domain binding 3 (LDB3) [32]. Additionally, RBM24 regulates alternative splicing as a splicing activator in the heart, and inactivation of RBM24 impairs normal heart development in mice and causes early death. In RBM24 mutant cardiomyocytes, sarcomere formation is almost abolished, which is consistent with another study showing the significance of RBM24 for sarcomere assembly and cardiac contractility in zebrafish [41,42]. The RNA sequencing data showed 68 genes are subject to RBM24-dependent splicing, which are involved in cardiogenesis, sarcomere formation, and pathogenesis of hypertrophic and dilated cardiomyopathy [41,42]. The FOX-family splicing factors were also implicated in heart disease. Downregulation of RBFOX1 was associated with heart failure in humans and mice. RBFox1 deficiency aggravates pressure overload-induced heart failure in the mice model. Splicing analysis showed that RBFox1 was indispensable for conserved splicing of transcription factor myocyte enhancer factor-2 (MEF2) family, and RBFOX1-dependent differential expression profile of MEF2 isoform affects cardiac hypertrophic gene expression. Interestingly, re-expression of RBFOX1 in pressure overload mice model ameliorated the disease state of cardiac hypertrophy [43]. Recently, it was shown that RBM20 regulates alternative splicing of titin and other essential cardiac genes in the heart [18,19,20]. Deficiency of Rbm20 causes DCM in rat models and mutations in RBM20 have been identified in human patients with severe DCM [18]. In addition, RBM20 expression level varies in patients with heart disease, and its expression correlates with the splicing of RBM20 target genes, which demonstrates RBM20 involvement in disease progression [20]. Since RBM20 is a newly identified splicing factor in muscle tissue and an emerging target for heart failure treatment, we will discuss the role of RBM20 in heart disease in the remaining sections.

## 3. RBM20 and Its Targets in the Heart

**Figure 1:**
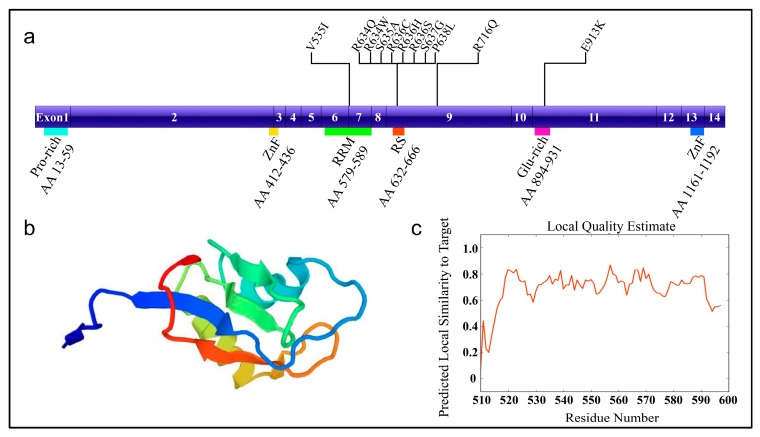
The schematic diagram of RNA-binding motif 20 (RBM20) with mutation sites. (**a**) RBM20 structure and known mutation sites. (**b**) Homologous protein structure modeled with SWISS-MODEL (Q5T481, *Homo sapiens*) [13]. Modeled region: AA 510-597, which contains RRM domain; QMEAN = −1.56 (QMENS value indicates the degree of nativeness of the structure features and whether the model is of similar quality to experimental structures; Scores of −4.0 or below indicates very low quality); (**c**) The local quality plot. For each residue of the model in Figure 1b (on the *x*-axis), *y*-axis shows the expected similarity of the model to the experimental structure. The residue showing a score below 0.6 are expected to be low quality. AA: amino acid; Pro-rich: proline-rich region; ZnF: zinc finger; RRM: RNA recognition motif; RS: arginine/serine-rich domain; Glu-rich: glutamic acid-rich region; V535I: valine (V) to isoleucine (I) change at AA position 535.

RBM20 has been recently cloned and is predominantly expressed in striated muscle, with the highest expression in the heart [19,20]. RBM20 also expressed in rat and human skeletal muscle, and almost no expression is found in non-muscle tissues. In 2008, Greaser an colleagues serendipitously found mutant rats with altered titin isoform expression identified by sodium dodecyl sulfate (SDS)-agarose gel electrophoresis [44,45]. These mutant rats finally divulged the secret of Rbm20. The RBM20 gene is located on human chromosome 10 and is composed of 14 exons encoding a large protein with a molecular weight of approximately 140 kDa. The RBM20 protein contains the typical domains commonly found in other splicing factors, which include a proline-rich region, a prototypical ribonucleic acid recognition motif (RRM), an arginine/serine-rich domain (RS), a glutamic acid-rich region, and U1 zinc finger domain (Figure 1) [18]. Both the RRM and RS domains of RBM20 is essential for nuclear retention of the protein, which is highly conserved between orthologues but does not share any similarities with other RS proteins [46,47]. A 95 kb deletion eliminating Rbm20 exons 2–14 can induce RBM20 splicing deficiency in rats, and generate mis-spliced target genes in the heart [18]. 

RBM20 regulates alternative splicing of crucial cardiac genes associated with sarcomere assembly, diastolic function and ion transport. RNA sequencing of the hearts of Rbm20-deficient rats and humans harboring a RBM20 mutation revealed 31 RBM20-genotype-dependent spliced genes, which were conserved between rats and humans (Table 2) [18]. In addition, a recent study identified 18 genes directly bound by RBM20 and subject to Rbm20-dependent alternative splicing in rat heart tissue (Table 2) [20]. Among these genes, eight genes including *Ttn*, *CaMKII-δ*, *Cacna1c*, *LDB3*, *Lmo7*, *Pdlim3*, *Rtn4* and *Ryr2* demonstrated conserved differential splicing of orthologous exons in humans and rats [18,20]. The exons in PEVK (Proline (P), Glutamate (E), Valine (V), Lysine (K)) and the immunoglobulin-rich region of titin were mis-spliced, which accounts for the dominant expression of the larger titin isoform and sarcomere distensibility in both Rbm20-deficient rats and humans harboring RBM20 mutations. RBM20 mutations cause exon 14 exclusion and exons 15 and 16 inclusion in CaMKII-δ. This aberrant splicing event induces an isoform switch from CaMKII-δB to CaMKII-δA. Also, RBM20 mutation causes changes in exon inclusion in calcium channel subunit Cacna1c, however, the effect is small. The aberrant splicing event in CaMKII-δ and Cacna1c can impact calcium homeostasis, and increase the risk of sudden death in RBM20 mutant species. For the LDB3 protein, RBM20 regulates differential inclusion of exon 4 (included in healthy humans or wild type (WT) rats) or exon 5 and 6 (included in the patient with RBM20 mutation or Rbm20 deficient rats), and the isoform switch of LDB3 has been related to DCM [18]. In addition, Rbm20 deficiency and mutations induce retention of exon 9 and 10 in Lmo7, which is a transcription factor essential for heart function. The Pdlim3 protein has two isoforms expressed in heart and skeletal muscle, respectively. The Rbm20 deficiency and mutation result in switching of the isoform to the skeletal muscle form that is associated with proper heart function. For Rtn4, Rbm20 deficiency and mutations induce exon 3 and 4 retention, but the function of Rtn4 in the heart is still unknown. In Ryr2, a 24 bp exon is upregulated in Rbm20 deficient rats and humans with RBM20 mutation, which also impacts the calcium homeostasis in the heart [20]. Taken together, mis-splicing of these orthologous exons by mutant RBM20 may induce abnormal biomechanics, electrical activity, and signal transduction. Finally, result in cardiomyopathy, arrhythmia and sudden death [18,20]. Remarkably, reduced expression of RBM20 has been identified in human heart failure influencing normal splicing of these target genes. This finding indicates a difference in expression level of RBM20 could also impact heart function [20]. The exact mechanism of how RBM20 regulates alternative splicing of these pivotal cardiac genes has not been determined, but the mechanism of RBM20-dependent titin alternative splicing is relatively well characterized [19,48].

## 4. RBM20-Dependent Alternative Splicing Mechanism

**Figure 2:**
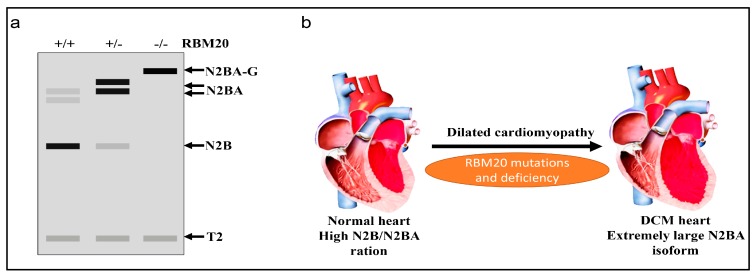
RBM20-dependent titin isoform expression. (**a**) Schematic representation of titin isoform expression in wild type (WT), Rbm20 heterozygous and Rbm20 homozygous rat heart analyzed by vertical agarose gel electrophoresis; N2BA-G, N2BA and N2B are different titin isoforms resulting from alternative splicing regulated by RBM20; T2 is a proteolytic fragment of titin; Grey box indicates low expression, Black box indicates high expression; (**b**) RBM20 mutations in humans and deficiency in rats lead to dilated cardiomyopathy (DCM).

**Figure 3:**
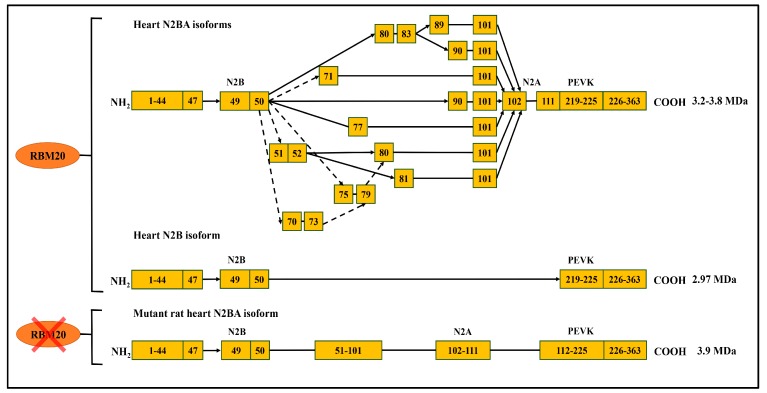
RBM20-dependant titin alternative splicing pathways. RBM20 regulates titin alternative splicing in the middle immunoglobulin (Ig) and PEVK (Proline (P), Glutamate (E), Valine (V), Lysine (K)) domain in the heart, however, the splicing events in PEVK region are not shown due to its complexity; without RBM20 in lower panel, most spliced exons indicated in the upper panel are spliced. Arrows represent exon spliced, while lines indicate consecutive exons (modified from [47]).

As discussed above, RBM20 is responsible for alternative splicing of various genes in the heart, and primarily regulates titin splicing [18,19,20]. Titin, a giant protein responsible for sarcomere integrity and passive elasticity of muscle, spans the sarcomere from the Z-line to the M-line. It has 364 exons located on human chromosome 2q31 [27]. The majority of the titin gene sequence is repetitive immunoglobulin (Ig) and fibronectin-3 regions, and the remaining part is a non-repetitive sequence composed of two unique sequences (N2B and N2A), PEVK (proline, glutamate, valine, lysine) region and a C-terminal Ser/Thr domain [48,49,50,51]. The Ig domain and the PEVK region experience vigorous alternative splicing [27,48]. With the combination of differentially spliced exons in these two regions, titin can produce millions of isoforms in striated muscles. However, only a few isoforms can be detected with current gel electrophoresis techniques, such as SDS-agarose gel and gradient sodium dodecyl sulfate polyacrylamide gel electrophoresis (SDS-PAGE) [48,52]. In WT adult rat heart, two major smaller isoforms, known as N2B and N2BA, can be observed with gel electrophoresis (Figure 2) [53,54,55,56]. WT Skeletal muscle expresses only one class of smaller isoform known as N2A. However, in Rbm20 deficient rats, a large titin isoform known as N2BA-G (approximately 3.9 MDa) is consistently expressed in striated muscle regardless of muscle type and developmental stage, and will eventually develop DCM (Figure 2) [18,19,56]. All these data indicate the pivotal role of RBM20 in titin splicing (Figure 3). 

In the middle Ig and PEVK region, the splicing patterns regulated by RBM20 have been recently studied. RBM20 acts as a splicing repressor and regulation is accomplished in a dose-dependent manner [19,20]. It was shown that RBM20 preferably binds to UCUU-containing RNA element in titin pre-mRNA and represses splicing of specific regions according to its sensitivity to RBM20 [19,20]. RBM20-repressed splicing occurs in two phases: i) RBM20 binds to the newly transcribed pre-mRNA and inhibits the removal of the introns in the bound regions. However, the rest of the pre-mRNA will be spliced. ii) the spliced pre-mRNA is localized and retained in the nucleus, after which 5’and 3’ splice sites on the exons flanking the RBM20-bound region are spliced together inducing the elimination of internal region [19]. In addition, a higher ratio of RBM20 to other splicing factors (serine/arginine-rich splicing factor 1 (SFRS1), U2AF65, heterogeneous nuclear ribonucleoprotein L (hnRNP L)) enhances repression activity of RBM20, which indicates the repression occurs in a dose dependent manner. For instance, the ratio of RBM20 to other splicing factors increases during development, while the titin size decreases [19]. 

Interestingly, recent studies demonstrated titin splicing could be regulated by hormones such as thyroid hormone and insulin in a RBM20-dependent manner [57,58]. Thyroid hormone could trigger titin isoform change in neonatal cardiomyocytes in an RBM20-dependent manner. Treatment with Triiodothyronine (T3) resulted in a decreased N2BA/N2B ratio, which could be induced by enhanced phosphorylation of RBM20 by protein kinase B (Akt) [58]. Also, insulin treatment activates the phosphoinositide 3-kinase-Akt-mammalian target of rapamycin (PI3K-Akt- mTOR) kinase pathway and increase the expression level of the N2B isoform with the presence of RBM20 [57]. Even though the precise role of RBM20 in the hormone-induced change in titin splicing is still not clear, these findings provide novel insights into developing therapeutic agents targeting RBM20 to induce titin isoform switching for treatment of heart disease [59].

## 5. RBM20 and Disease Origin in Induced Pluripotent Stem Cells

Most of the studies concerning RBM20 have been conducted with the rat model and human heart tissue. The largest obstacle to study the molecular and cellular physiology of the heart is the difficulty maintaining functional human cardiomyocytes in culture [60]. However, recent studies applied patient-specific induced pluripotent stem cell (iPSCs) derived cardiomyocytes to recapture the human heart disease phenotype in vitro [61]. These studies showed RBM20 mutation or RBM20 knockdown in pluripotent stem cells is sufficient to recapitulate molecular defects in patients with DCM [60,62,63]. Also, these findings are consistent with previous Rbm20-deficient rat model exhibiting structural and functional defects in the heart. Notably, studies not only showed splicing of RBM20 but also demonstrated RBM20-dependent transcriptional regulation [60,62,63,64]. Some of the changes in gene expression have been previously identified as the primary effect of RBM20 inactivation including myosin heavy chain 7 (MYH7) and myomesin 1 (MYOM1). However, whether the changes in expression of other genes directly caused by RBM20 inactivation or not remains to be further confirmed.

One recent study generated iPSC cardiomyocytes (iPSC-CMs) from a DCM patient harboring a missense mutation S635A in RBM20. The RBM20 mutant-iPSC-CMs showed the abnormal distribution of α-actinin and calcium mishandling compared to the healthy group. In addition, engineered heart muscle derived from RBM20 mutant-iPSC-CMs exhibited a decrease in both active force production and passive stress of the muscle. RBM20 mutant-iPSC-CMs showed a reduction in the expression level of the N2B titin isoform and exon skipping events in the PEVK region. Transcriptome profile revealed that RBM20 mutant-iPSC-CMs differentially expressed 161 genes (DEGs) compared to the control. The most regulated DEGs are related to cellular component as stress fiber (*MYLK*, *TEK*, *UNC13D*), negative regulation of protein depolymerization (*FGF13*, *MID1*, *SCIN*, *SHROOM2*), drug metabolism (*ADH1B*, *GSTT2*, *UGT2B15*), intercalated disc (*DES*, *DSC2*, *DSG2*, *FGF13*), glycosaminoglycan metabolic processes (*BGN*, *CSGALNACT1*, *CYTL1*, *EGFLAM*, *FGF13*, *GPC3*, *GPC4*, *HS6ST2*) and response to amino acids (*COL4A6*, *CYP21A2*, *DNMT3B*, *FOLR1*, *ICAM1*, *MMP3*, *MUC1*). This study identified 34 genes with changes in exon usage, but the overlap between spliced and expressed genes is little. These data revealed that differential gene expression and alternative splicing could be associated with distinct signaling pathways. Significant changes have been found in exon usage of novel genes including genes associated with cardiac muscle tissue development (COL14A1), myosin complex (MYL7), ribosomal small subunit biogenesis (RSP28, RPS6), virion assembly (DDX6, UBCV), and regulation of tumor necrosis factor-mediated signaling pathway (TNFRSF1A, UBC) [60]. Another study used iPSC-CMs derived from the patient with an RBM20 R636S missense mutation. They found abnormal splicing affecting isoform changes in certain genes as previously described. Also, the microarray analysis showed 50 genes differentially expressed, among which 38 genes were downregulated, and 12 genes were upregulated. The gene ontology enrichment assay related function of these 50 genes to pattern specification processes, embryonic heart tube development, response to the metal ion, embryonic organ development and cholesterol homeostasis [63]. Recently, tnnt2-pGreenZeo pluripotent stem cells were engineered to silence Rbm20 expression. In 12 day-Rbm20-deficient mice embryos, 49 genes were differentially spliced, and 21 genes were differentially expressed compared to the control group. Most of the differentially expressed genes were related to heart development and muscle differentiation [62].

In addition, one study showed pharmacological modulation of calcium homeostasis in RBM20 patient-derived iPSC model. They found β-adrenergic treatment in RBM20 mutant-iPSC-CMs would exacerbate calcium mishandling, apoptotic changes and sarcomere disarray. However, pretreatment with β-blockers, carvedilol, verapamil or calcium channel blocker ameliorated the pathogenic phenotype after beta-adrenergic stimulation [65]. Altogether, these studies provided robust functional genomic or pharmaceutical insights into RBM20 mutant and deficient DCM models, which can help to develop patient-specific therapeutic agents and strategies in the future. 

## 6. RBM20 Mutations and Heart Disease

The function of different muscle splicing factors is relatively well understood compared to RNA-binding proteins, whose role in normal and diseased heart have been recently demonstrated. Few cases concerning the mutations in muscle-specific factors have been related to cardiomyopathies. Until recently, only mutations in RBM20 has been linked to heart disease [21,22]. Mutations in RBM20 are responsible for 3% to 5% of all familial DCM cases [22,66]. The most common mutations in human RBM20 include mutations in the RRM domain (V535I), and in the RS domain (R634Q, R634W, S635A, R636C, R636H, R636S, S637G, P638L) [21,22,67]. Another two mutations outside of the mutation hot spot have also been identified: one mutation in exon 9 causing R716Q and the other in the glutamate-rich region causing E913K (Figure 1) [22,23]. These mutations possibly lead to RBM20 protein misfolding and interfere with its ability to bind the target RNA sequence [20]. As evidenced by previous studies, these mutations can induce titin isoform switching to the highly compliant N2BA isoform [18,19,21]. Alternations in splicing or expression of essential cardiac genes in humans harboring RBM20 mutations and patient-specific iPSC-CM are described in the previous sections, all of which account for the pathogenesis of RBM20 mutation induced DCM.

## 7. Future Directions

Development of RNA biology has led to the discovery of novel muscle-specific splicing factors and their function. The knowledge of the involvement of disrupted splicing factors and splice isoforms in heart disease is continuously evolving. The RBM20 associated heart disease is mainly based on the mis-splicing of pivotal cardiac genes, among which titin is recognized as a major human disease gene [18,20,68]. The titin-dependent passive tension in the heart can be manipulated in two ways either by alternative splicing or post-transcriptional modification [69,70,71]. Since it is known that RBM20 mainly regulates titin alternative splicing, future work can focus on developing therapeutic agents targeting RBM20 to shift titin isoform in heart disease. For instance, inhibition of RBM20 improves diastolic function in a mouse model of heart failure and reducing RBM20 can ameliorate cardiac atrophy [72,73,74]. Since RBM20 can target multiple genes, targeting upstream signals of RBM20 to modulate RBM20 and thus downstream genes would be a therapeutical strategy. Another aspect that should be considered is that RBM20 is a multi-functional splicing factor. In human iPSC-CMs, RBM20 mutations induce both differential exon usage and differential gene expression of essential cardiac genes [60,62,63]. However, how RBM20 induces up or downregulation of cardiac genes is still unclear. In addition, RNA binding proteins are involved in the biogenesis of circular RNAs (circRNAs), a new type of non-coding RNAs. Recent reports indicated that around eighty circRNAs were expressed from the titin gene and that the expression of some of these circRNAs is dynamically regulated in DCM but not in hypertrophic cardiomyopathy (HCM). Further, RBM20-null mice display a complete lack these titin circRNAs. This finding indicated a function of RBM20 in back splicing of titin RNA, and that RBM20 deficiency triggers DCM not only by mis-splicing of certain cardiac genes but also by eliminating circRNAs production from titin gene [75]. Taken together, future work should be focused on understanding the multi-functional role of RBM20 and developing therapeutic agents targeting RBM20 in heart diseases.

## Figures and Tables

**Table 1:** Muscle-specific splicing factors and heart disease.

Splicing Factors	Disease Association	Target Genes	References
CUGBP 1	Myotonic dystrophy Cardiac conduction abnormalities	*TNNT2* and *LDB3*	[30,31,32,33,34]
MBNL 1	Myotonic dystrophy Cardiac conduction abnormalities	*TNNT2* and *LDB3*	[34,35,36,37,38,39,40]
RBM24	Hypertrophic and dilated cardiomyopathy	*TNNT2*, *TPM1*, *TPM2*, *ACTB* and other 68 genes	[41,42]
RBFOX1	Cardiac hypertrophy and heart failure	*NRAP*, *MYBPC3*, *LDB3*	[43]
RBM20	Dilated cardiomyopathy	TTN *LDB3* and other 29 genes	[18,19,20]

Troponin T2 (*TNNT2*); LIM domain binding 3 (*LDB3*); Titin (*TTN*); Tropomyosin 1 (*TPM1*); Tropomyosin 2 (*TPM2*); Actin beta (*ACTB*); Nebulin-related anchoring protein (*NRAP*); Myosin binding protein C, cardiac (*MYBPC3*).

**Table 2:** Conserved set of genes with RBM20-dependent alternative splicing in humans and rats, and direct Rbm20-regulated genes in rat heart.

Species Specificity	Gene Symbol	Name	Associated Heart Disease	Reference
Conserved set of genes with RBM20-dependent alternative splicing in humans and rats	*APTX*	Aprataxin	NA	[18]
	*Cacna1c*	Calcium Voltage-Gated Channel Subunit α1 C	Heart failure	[18]
	*CaMKII-*δ	Calcium/calmodulin dependent protein kinase II δ	Heart failure, DCM	[18]
	*CAMKIIG*	Calcium/calmodulin dependent protein kinase II gamma	Heart failure	[18]
	*DAB1*	DAB1, reelin adaptor protein	NA	[18]
	*DNM3*	Dynamin 3	NA	[18]
	*DTNA*	Dystrobrevin alpha	DCM	[18]
	*FHOD3*	Formin homology 2 domain containing 3	NA	[18]
	*FNBP1*	Formin binding protein 1	NA	[18]
	*GIT2*	GIT ArfGAP 2	Heart failure	[18]
	*KALRN*	Kalirin RhoGEF kinase	NA	[18]
	*KCNIP2*	Potassium voltage-gated channel interacting protein 2	Heart failure, DCM	[18]
	*LDB3*	LIM domain binding 3	DCM	[18]
	*MECP2*	Methyl-CpG binding protein 2	NA	[18]
	*MTMR1*	Myotubularin related protein 1	NA	[18]
	*NFIA*	Nuclear factor I A	NA	[18]
	*NPRL3*	NPR3 like, GATOR1 complex subunit	NA	[18]
	*NTRK3*	Neurotrophic receptor tyrosine kinase 3	NA	[18]
	*PDLIM5*	PDZ and LIM domain 5	NA	[18]
	*PLEKHA5*	Pleckstrin homology domain containing A5	NA	[18]
	*RALGPS1*	Ral GEF with PH domain and SH3 binding motif 1	NA	[18]
	*SEMA6D*	Semaphorin 6D	NA	[18]
	*SH3KBP1*	SH3 domain containing kinase binding protein 1	NA	[18]
	*SLC38A10*	Solute carrier family 38 member 10	NA	[18]
	*SPEN*	Spen family transcriptional repressor	NA	[18]
	*SORBS1*	Sorbin and SH3 domain containing 1	NA	[18]
	*TRDN*	Triadin	NA	[18]
	*TPM1*	Tropomyosin 1	Heart failure, DCM	[18]
	*Ttn*	Titin	Heart failure, DCM	[18]
	*UBE2F*	Ubiquitin conjugating enzyme E2 F	NA	[18]
	*ZNF451*	Zinc finger protein 451	NA	[18]
Unique direct RBM20-regulated genes in rat heart	*DST*	Dystonin	NA	[20]
	*ENAH*	Enabled homolog	NA	[20]
	*IMMT*	Inner membrane protein, mitochondrial	NA	[20]
	*LMO7*	LIM domain only protein 7	NA	[20]
	*MLIP*	Muscular-enriched A type laminin-interacting protein	NA	[20]
	*LRRFIP1*	Leucine-rich repeat interacting protein 1	NA	[20]
	*MYH7*	Myosin heavy chain 7	NA	[20]
	*MYOM1*	Myomesin 1	NA	[20]
	*NEXN*	Nexilin	NA	[20]
	*OBSCN*	Obscurin	NA	[20]
	*PDLIM3*	PDZ and LIM domain 3	NA	[20]
	*RTN4*	Reticulon 4	NA	[20]
	*RYR2*	Ryanodine receptor 2	NA	[20]
	*TNNT2*	Troponin T type 2	NA	[20]
